# Supplementary material for: A Pilot Study on a Nurse Rehabilitation Program: Could It Be Applied to COVID-19 Patients?
Source: Int J Environ Res Public Health. 2022 Nov 2;19(21):14365. doi: 10.3390/ijerph192114365 (PMC9654829; doi:10.3390/ijerph192114365)
Supplement: Supplementary file 1 [file ijerph-19-14365-s001.zip › ijerph-1975150-supplementary.pdf]

International Journal of
Environmental Research and Public Health

MDPI

Article

A Pilot Study on a Nurse Rehabilitation Program: Could It Be Applied to COVID-19 Patients?

Supplementary Materials

Table S1. Details of the checklist.

Teaching	Understand		Performs	
	**Yes**	**No**	**Effective**	**No Effective**
Rest and relaxation techniques				
Breathing awareness and control				
Exhalation with pursed lips, with prolongation of the expiratory time				
Incentive spirometry				
Global costal opening				
Diaphragmatic reeducation with resistance				
Cough				
Energy conservation measures				
